# Intracellular targeting of Cisd2/Miner1 to the endoplasmic reticulum

**DOI:** 10.1186/s12860-021-00387-1

**Published:** 2021-09-30

**Authors:** Claudie Bian, Anna Marchetti, Philippe Hammel, Pierre Cosson

**Affiliations:** 1 Department of Cell Physiology and Metabolism, Faculty of Medicine, University of Geneva, 1 rue Michel Servet, 1211 Geneva 4, Switzerland; 2 Manufacturing Science and Technologies, Biotech Department, Merck, Z.I. de l’Ouriettaz 150, 1170 Aubonne, Switzerland

**Keywords:** CISD1, CISD2, mitoNEET, Miner1, Secretory pathway, Mitochondria, Transmembrane domain, Dilysine motif, COPI, Endoplasmic reticulum

## Abstract

**Background:**

Cisd1 and Cisd2 proteins share very similar structures with an N-terminal membrane-anchoring domain and a C-terminal cytosolic domain containing an iron-cluster binding domain and ending with a C-terminal KKxx sequence. Despite sharing a similar structure, Cisd1 and Cisd2 are anchored to different compartments: mitochondria for Cisd1 and endoplasmic reticulum for Cisd2. The aim of this study was to identify the protein motifs targeting Cisd2 to the ER and ensuring its retention in this compartment.

**Results:**

We used new recombinant antibodies to localize Cisd1 and Cisd2 proteins, as well as various protein chimeras. Cisd2 is targeted to the ER by its N-terminal sequence. It is then retained in the ER by the combined action of a C-terminal COPI-binding KKxx ER retrieval motif, and of an ER-targeting transmembrane domain. As previously reported for Cisd1, Cisd2 can alter the morphology of the compartment in which it accumulates.

**Conclusion:**

Although they share a very similar structure, Cisd1 and Cisd2 use largely different intracellular targeting motifs to reach their target compartment (mitochondria and endoplasmic reticulum, respectively).

**Supplementary Information:**

The online version contains supplementary material available at 10.1186/s12860-021-00387-1.

## Background

The CDGSH domain binds (Fe_2_S_2_) iron-sulfur clusters. It is found in three human proteins: Cisd1 (also referred to as mitoNEET), Cisd2 (Miner1; NAF-1) and Cisd3 (Miner2). Cisd1 and Cisd2 are the most studied members of the family and have a very similar organization: their N-terminal portion contains a transmembrane domain (TMD), and the CDGSH domain is located in the cytosol, where it forms a dimer. Cisd1 is anchored by its N-terminus in the outer membrane of mitochondria [1]. The situation is less clear for Cisd2 which has been proposed to be anchored to the endoplasmic reticulum (ER) [1–3] or to mitochondria [4].

While the presence of a CDGSH domain in Cisd proteins clearly suggests that they play a role in iron homeostasis, their precise molecular function is still a matter of investigation. CISD1 may serve as a sensor of oxidative signals in cells [5], and may regulate cell death and proliferation, particularly in cancer cells [6]. On the other hand, mutations in *CISD2* can be the cause of Wolfram syndrome, a rare genetic neurodegenerative disease, presumably caused by ER stress [7]. Genetic inactivation of *CISD2* in mouse embryonic fibroblasts resulted in structural and functional alterations of both the ER and mitochondria, induction of the ER unfolded protein response, a decrease in ER calcium concentration and an increase in mitochondrial calcium concentration [3]. In mice, disruption of *CISD2* caused mitochondrial alterations, increased autophagy and cell death, and reduced longevity of the animals [4]. Disruption of *CISD2* was also observed to increase autophagy in cultured H1299 epithelial cells and this was proposed to be mediated by an interaction between Cisd2 and Bcl2 [2].

In addition to its proposed role in iron metabolism and oxygen sensing, Cisd1 may play a structural role in cells by tethering mitochondria with one another. Indeed, overexpression of Cisd1 increased tethering of mitochondria and caused their aggregation, while genetic inactivation of *CISD1* decreased mitochondrial tethering [8]. This probably reflects the fact that the Cisd1 cytosolic domains form homodimers, so that two molecules anchored to neighboring mitochondria can establish stable bridges between the two membranes. It remains to be seen if Cisd2 can play a similar role in the ER.

The presence of a KKEV sequence at the C-terminus of Cisd2 was noted previously and it was proposed that it could act as a canonical ER-targeting motif [2]. Indeed C-terminal KKxx sequences can ensure ER localization by binding COPI cytosolic complexes and targeting proteins for continuous retrieval from the Golgi apparatus to the ER [9, 10]. However, the role of the Cisd2 KKEV sequence was not investigated. Surprisingly, a similar motif (KKET) is found at the C-terminus of Cisd1. This observation is difficult to reconcile with the fact that Cisd1 is localized in the membrane of mitochondria, and its significance remains unclear.

Our aim in this study was to establish how Cisd1 and Cisd2 are targeted to mitochondria and the endoplasmic reticulum, respectively. In addition, we wished to determine if Cisd2, like Cisd1, has the ability to alter the overall organization of the compartment(s) in which it is present.

## Results

### The N-terminal region of Cisd1 and Cisd2 targets them to mitochondria and the endoplasmic reticulum, respectively

**Fig. 1:**
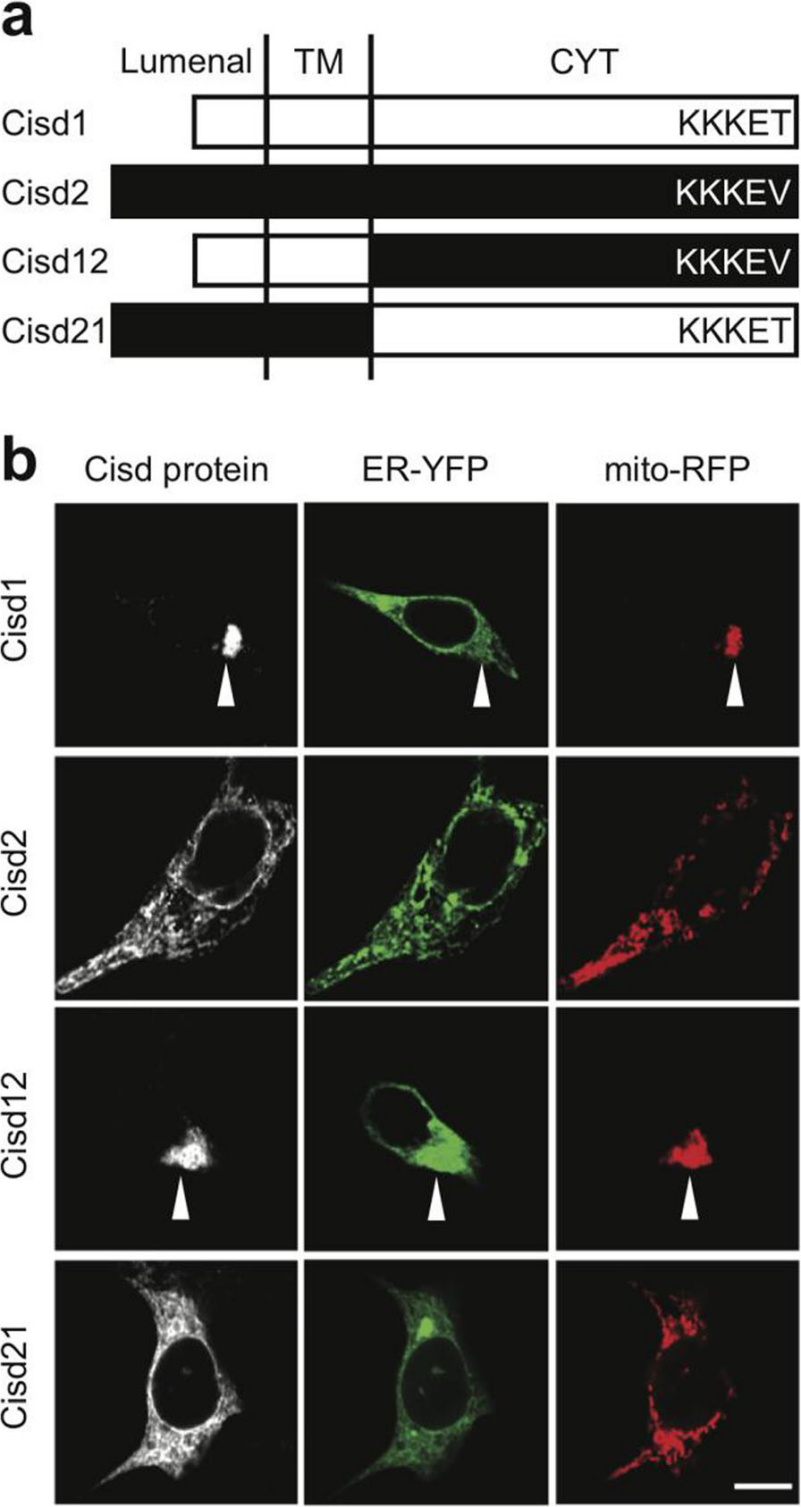
The N-terminal portion of Cisd2 targets it to the ER. **a** Schematic representation of natural and chimeric Cisd1 and Cisd2 proteins. The Cisd12 (respectively Cisd21) protein is composed of the N-terminal portion of Cisd1 (respectively Cisd2) attached to the Cisd2 (respectively Cisd1) cytosolic domain. The detailed amino acid sequence of these proteins is shown in Additional file 4. **b** Immunofluorescence localization of Cisd proteins. HEK cells were co-transfected to produce the indicated Cisd protein, ER-targeted YFP and mitochondria-targeted RFP. Immunofluorescence staining was performed using specific antibodies against Cisd1 or Cisd2 cytoplasmic domain. All pictures were taken with a confocal microscope (LSM700, Zeiss). Scale bar: 10 μm. The arrowheads point to aggregates of mitochondria. A second panel of pictures is shown in Additional file 5

The primary targeting of proteins during or shortly after their translation is often controlled by their N-terminal sequence. In both Cisd1 and Cisd2, a hydrophobic segment is present in the N-terminal portion (Fig. 1a). Accordingly, this region of the protein may target CISD1 and CISD2 to the ER or to mitochondria, or to both compartments. In order to verify this hypothesis, we first produced in HEK cells Cisd1 or Cisd2 together with a yellow-shifted green fluorescent protein targeted to the ER (ER-YFP) and a red fluorescent protein targeted to mitochondria (mito-RFP) (Fig. 1b). Importantly, we expressed native Cisd1 and Cisd2 proteins. Indeed, fusion to additional sequences (peptide tags or fluorescent proteins) facilitates detection of proteins, but may alter their intracellular localization. To avoid potential artefacts, we developed recombinant antibodies recognizing specifically either the Cisd1 cytosolic domain (RB251) [11] or the Cisd2 cytosolic domain (RB253) [12, 13]. RB251 detected the presence of endogenous Cisd1 in HEK cells (Additional file 1). On the contrary RB253 did not detect significant amounts of Cisd2 in HEK cells (Additional file 1). The signal generated by RB253 in untransfected HEK cells was so weak that we cannot unambiguously determine if it corresponds to a non-specific binding of the antibody to cellular structures, or to the specific detection of a very small amount of endogenous Cisd2. Each antibody detected a large amount of its target protein in cells transfected with adequate expression plasmids (Additional file 1). As expected, we observed that in transfected cells, Cisd1 was localized in mitochondria, and absent from the ER (Fig. 1b). In addition, as previously reported [8], overexpression of Cisd1 resulted in the aggregation of mitochondria (Fig. 1b, arrowhead). On the contrary, Cisd2 was clearly colocalized with ER-targeted YFP, in particular at the level of the nuclear envelope, and not detected in mitochondria (Fig. 1b). We cannot exclude that a small portion of Cisd2 was localized in mitochondria, but then its concentration would be much weaker than in the ER. The structure of the ER often appeared slightly altered in cells expressing Cisd2 (Fig. 1b): the ER was less finely dispersed in the cytosol, and the nuclear envelope was less regularly organized. However, the limited resolution of immunofluorescence microscopy and the significant cell-to-cell variability prevented us from ascertaining this point unambiguously. We made almost identical observations in HEK cells and in three other cell types (Hela, HCT116 and Huh–7): endogenous Cisd1 is localized to mitochondria and its overexpression induces clustering of mitochondria (Additional file 2). Endogenous Cisd2 is undetectable. In transfected cells it is localized in the ER and perturbs its structure (Additional file 3).

We then produced in HEK cells a chimeric protein (Cisd12) composed of the N-terminal portion of Cisd1, fused to the cytosolic region of Cisd2 (Fig. 1a; Additional file 4). Cisd12 was clearly targeted to mitochondria, and remarkably, like Cisd1 it was capable of causing aggregation of mitochondria (Fig. 1b, arrowhead). We also produced a chimeric protein (Cisd21) composed of the N-terminal portion of Cisd2, fused to the cytosolic domain of Cisd1 (Fig. 1a) and observed that it was localized in the ER. Since there is a significant cell-to-cell variability in the morphology of the ER and of mitochondria, a second set of pictures is presented, essentially confirming the results shown in Fig. 1b (Additional file 5). Together these results demonstrate that the N-terminal region of Cisd1 and Cisd2 ensures their targeting to the mitochondria and to the ER, respectively.

### Cisd2 is retained in the ER by its TMD and its cytosolic KKxx motif

**Fig. 2:**
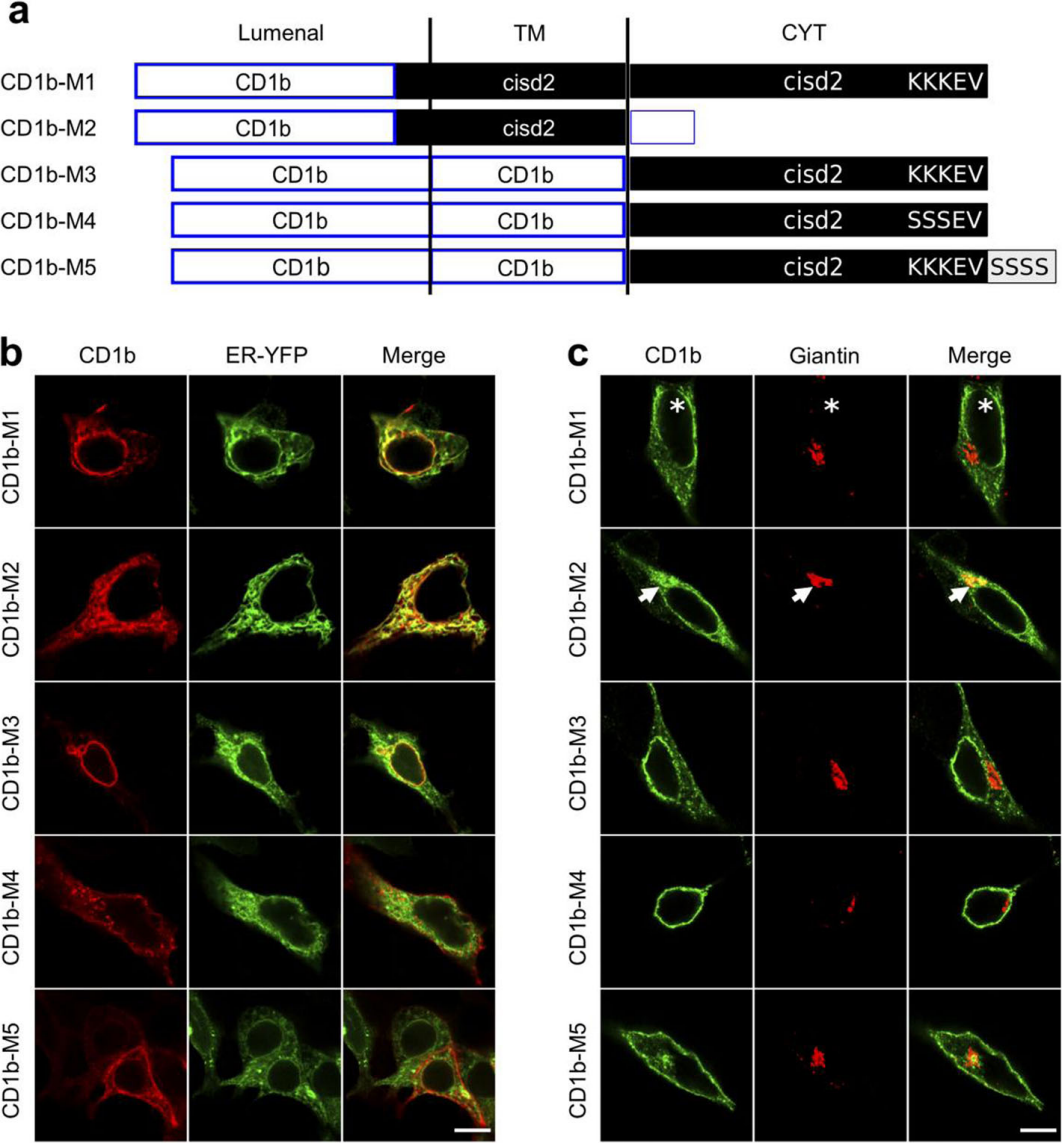
The TMD and the cytosolic KKEV motif of Cisd2 jointly ensure its efficient localization in the early secretory pathway. **a** Schematic representation of CD1b-Cisd2 fusion proteins. Various portions of the Cisd2 protein were fused with the CD1b extracellular domain and TMD. In some constructs, the C-terminal KKEV motif was mutated either by replacing three lysine residues with serine residues, or by adding four serine residues at the C-terminal extremity. The detailed amino acid sequence of these proteins is shown in Additional file 6. **b** Colocalization of CD1b-Cisd2 fusion proteins with an ER marker. HEK cells co-expressing the indicated CD1b-Cisd2 chimera and ER-YFP were labeled by immunofluorescence, using an anti-CD1b antibody. CD1b-M1, 2 and 3 were largely localized in the ER, CD1b-M4 and 5 were not. **c.** Colocalization of CD1b-Cisd2 fusion proteins with the Golgi apparatus. HEK cells expressing the indicated proteins were labeled by immunofluorescence, using an anti-CD1b antibody. The Golgi complex was revealed with an anti-giantin antibody. All pictures were taken with a confocal microscope (LSM700, Zeiss). Scale bar: 10 μm. The asterisks indicate a nucleus with a discontinuous staining of the nuclear envelope, the arrows point to a Golgi apparatus. A second panel of pictures is shown in Additional file 7

Our next aim was to determine how Cisd2, once inserted in the ER, avoids being transported to the cell surface with the bulk of membrane proteins. In order to separate co-translational insertion in the ER from later intracellular transport events, and to provide an invariant method to detect various mutant proteins, we fused Cisd2 at its N-terminal end with a signal sequence and the coding sequence of the luminal domain of CD1b (Fig. 2a; Additional file 6). The resulting CD1b-M1 protein was produced in HEK cells and largely colocalized with ER-targeted YFP, in particular in the nuclear envelope (Fig. 2b). Similar to cells expressing Cisd2, the fine structure of the ER appeared altered in some cells expressing CD1b-M1 (Fig. 2b). In some cells the structure of the nuclear envelope was profoundly modified, with CD1b-M1 unevenly distributed around the nucleus (Fig. 2c; asterisks). For all CD1b chimeric proteins, a duplicate set of images is provided in Additional file 7.

**Fig. 3:**
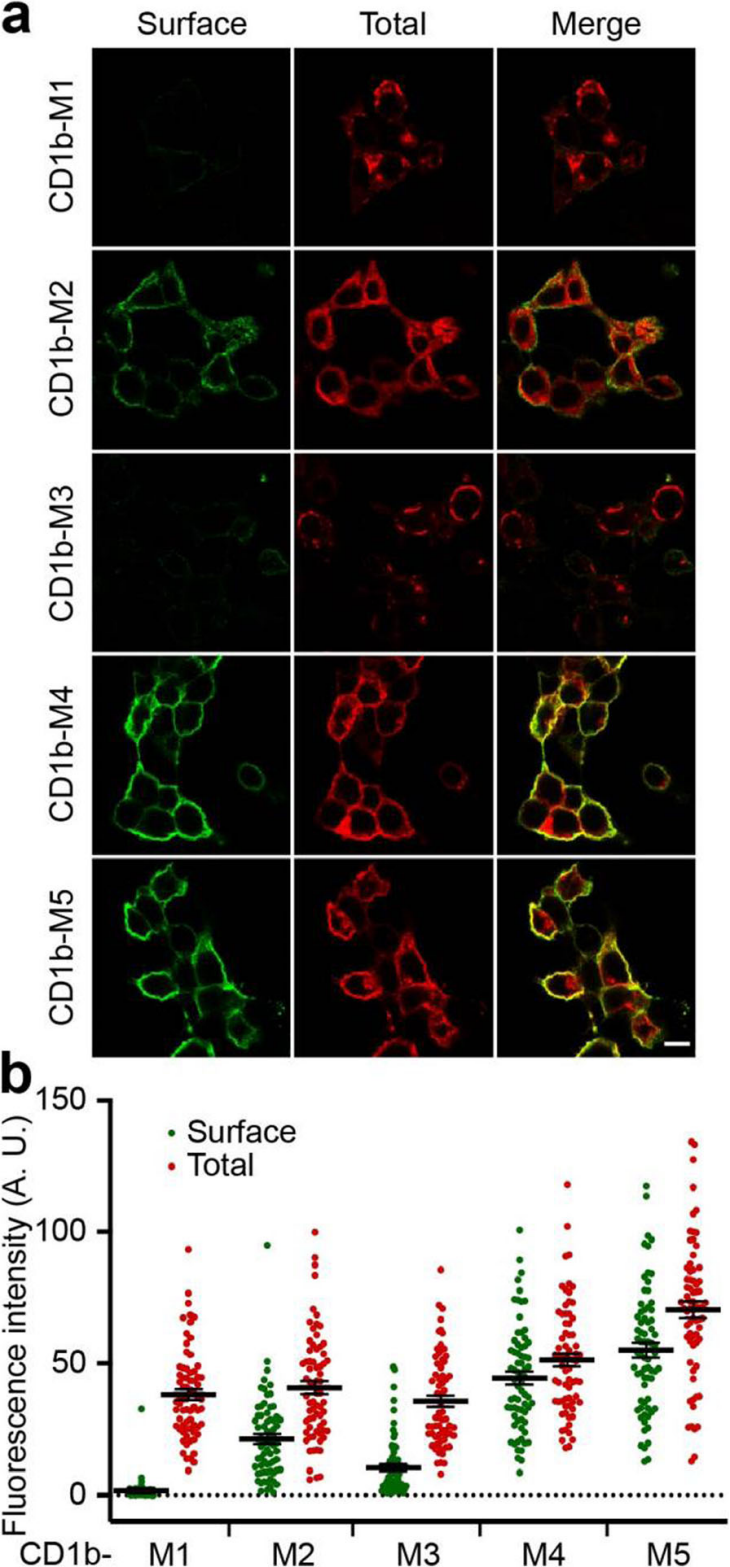
The TMD and the cytosolic KKEV motif of Cisd2 cooperate to limit its surface expression. **a** HEK cells expressing the indicated CD1b-Cisd2 fusion proteins were labeled by immunofluorescence before (Surface) and after (Total) permeabilization, using antibodies specific for the CD1b extracellular domain. All pictures were taken with the same settings with a confocal microscope (LSM700, Zeiss). Scale bar: 10 μm. **b** The amount of each CD1b fusion protein detected at the cell surface and in the whole cell was quantified. Each dot represents the fluorescence measured in one individual cell. CD1b-M1 was absent from the cell surface, small but significant levels of CD1b-M2 and CD1b-M3 were detected at the cell surface. CD1b-M4 and CD1b-M5 accumulated largely at the cell surface

Two elements could in principle ensure efficient ER retention of CD1b-M1: its TMD and its C-terminal KKxx sequence. The TMD of Cisd2 is unusually short (16AA), a feature which can ensure ER retention of some transmembrane proteins [14]. To test this hypothesis, we produced in HEK cells a chimeric protein composed of the CD1b extracellular domain fused to the TMD of Cisd2, and a short cytosolic domain (CD1b-M2). CD1b-M2 was mostly localized in the ER (Fig. 2b), and also to the Golgi apparatus where it was colocalized with giantin, a marker of the Golgi apparatus (Fig. 2c; arrow). We also tried to detect the presence of CD1b fusion proteins by surface immunolabeling. While CD1b-M1 was virtually absent from the cell surface, a significant fraction of CD1b-M2 was detected at the cell surface (Fig. 3). These results indicate that the TMD of Cisd2 does confer ER retention, but not as efficiently as the full Cisd2 protein.

The second element that may ensure ER localization of Cisd2 is the KKEV sequence found at its C-terminus. In order to test this hypothesis, we first produced a CD1b-M3 fusion protein composed of the CD1b extracellular and TMD fused to the cytosolic domain of Cisd2 (Fig. 2a). The CD1b-M3 protein was mostly localized in the ER (Fig. 2b), with no apparent colocalization with a Golgi marker (Fig. 2c). However, a small but significant amount of CD1b-M3 was detected at the cell surface (Fig. 3). In order to ascertain whether ER localization of CD1b-M3 was due to the presence of a C-terminal KKxx motif, we disrupted the putative KKxx motif by mutating its two lysine residues to serine residues (CD1b-M4; Fig. 2a) or by adding 4 additional serine residues at the C-terminal end of Cisd2 (CD1b-M5; Fig. 2a). Both CD1b-M4 and CD1b-M5 were poorly retained in the ER or the Golgi apparatus (Fig. 2b and c), and mostly present at the cell surface (Fig. 3). These conclusions were confirmed by western blot analysis of the CD1b chimeric proteins (Additional file 8): only CD1b-M1, CD1b-M4 and CD1b-M5 showed high molecular weight species typical of proteins bearing mature sugars, indicating that they have escaped massively the ER to reach the plasma membrane.

Overall, these results indicate that the efficient ER localization of CD1b is achieved by the additive effect of its short TMD and its C-terminal cytosolic KKxx motif.

### The Cisd2 KKEV binds COPI, Cisd1 KKET does not

**Fig. 4:**
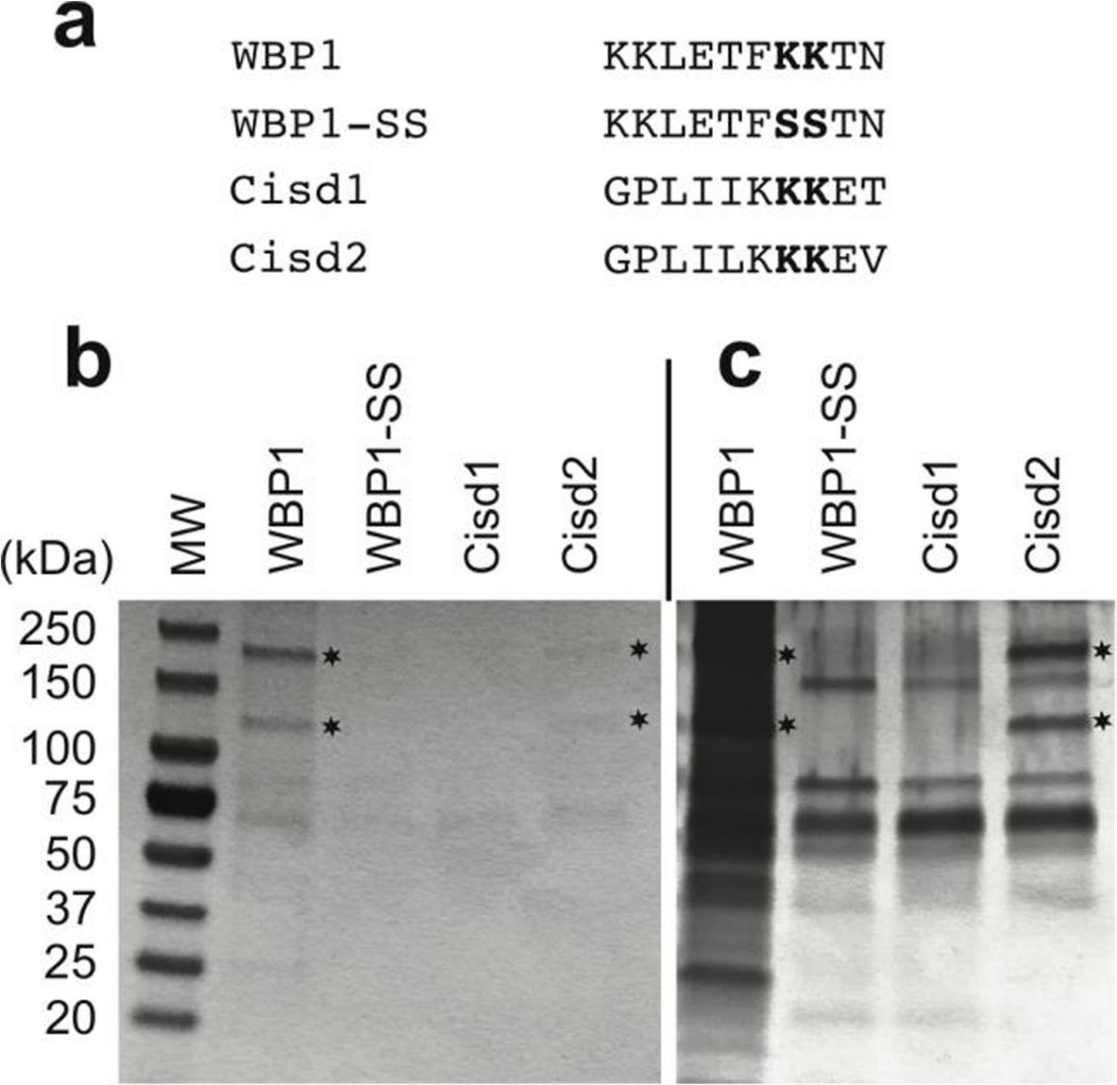
The Cisd2 KKEV motif of Cisd2 interacts with the COPI complex. **a** Synthetic peptides corresponding to the cytosolic C-terminal sequence of Cisd1, Cisd2 and the yeast WBP1 protein were synthesized and biotinylated. As a negative control, we used a peptide where the two lysine residues of WBP1 were replaced with two serine residues (WBP1-SS). **b** The indicated peptides were immobilized on beads and incubated with a lysate from COS7 cells. The proteins bound to the peptides were then separated on an acrylamide gel and detected by Coomassie staining. The position of the α (≈160 kDa) β, β’ and γ (≈110 kDa) subunits of the COPI complex are indicated with stars. **c** The gel shown in b was restained with silver to detect less abundant proteins. The COPI complex interacted specifically with the WBP1 cytosolic domain, as well as with the Cisd2 cytosolic domain

Although the presence of a functional KKEV ER retrieval motif in Cisd2 seems relevant, the presence of a very similar sequence (KKET) in mitochondrial Cisd1 is more surprising. To investigate this point, we assessed the ability of the Cisd1 and Cisd2 C-terminal regions to bind cytosolic COPI. For this, peptides corresponding to the 10 C-terminal residues of Cisd1 and Cisd2 were synthesized and coupled to avidin at their N-terminal extremity (Fig. 4a). For comparison we used the well-characterized WBP1 KKxx COPI-binding motif and a mutant (WBP1-SS) unable to bind COPI [9]. As previously observed [9], when cell lysates were incubated with these peptides, COPI bound to WBP1 but not to WBP1-SS (Fig. 4b). The biggest subunits of the COPI complex (α: 170 kDa; β, β’ and γ: 110 kDa) were clearly detected by Coomassie blue staining (Fig. 4b; stars). COPI also bound, though less efficiently, to the Cisd2 peptide: COPI subunits were barely detectable in a Coomassie-stained gel (Fig. 4b), but readily apparent in a silver-stained gel (Fig. 4c). COPI did not visibly bind the Cisd1 peptide. These observations confirm the fact that Cisd2 presents a functional COPI-binding KKxx motif. On the contrary, despite the high degree of sequence similarity, the Cisd1 C-terminus does not have the ability to bind efficiently the COPI complex.

### Cisd2 expression alters the structure of the ER

As detailed above, immunofluorescence analysis suggested that expression of Cisd2 alone or fused to the CD1b extracellular domain (CD1b-M1) altered the structure of the ER and of the nuclear envelope. However, the limited resolution of light microscopy and the heterogenous morphology of the ER prevented us from drawing firm conclusions.

**Fig. 5:**
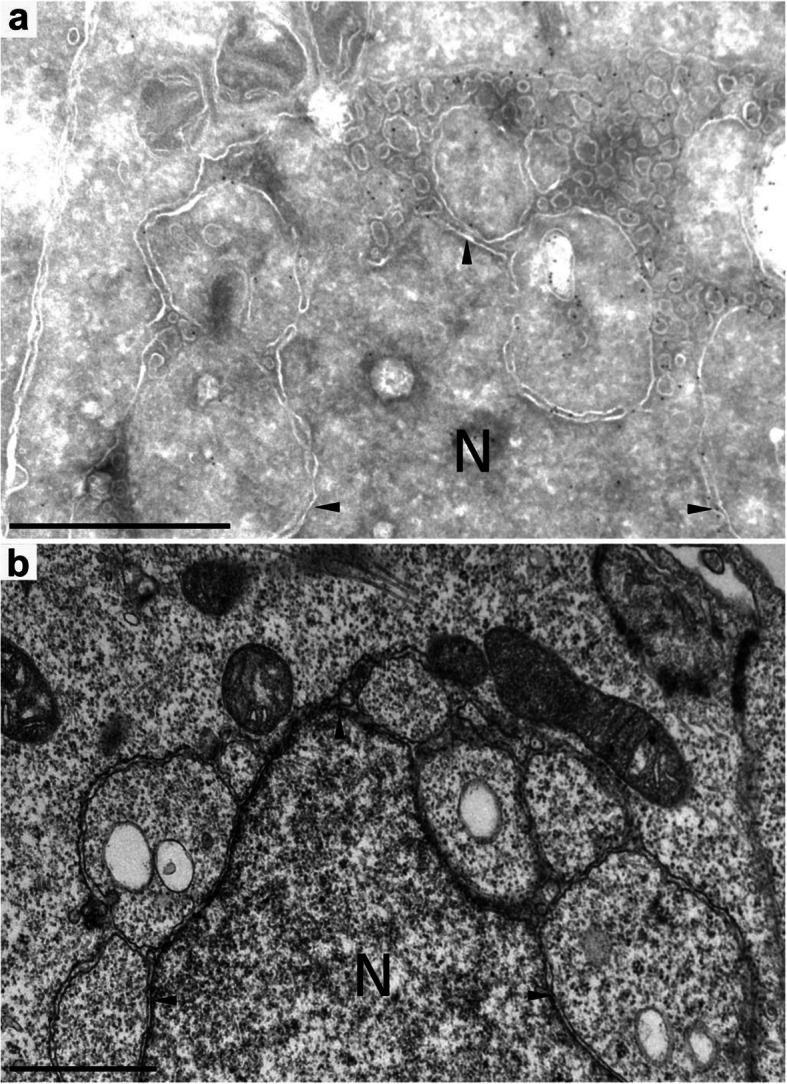
Expression of CD1b-tagged Cisd2 alters the morphology of the endoplasmic reticulum. **a** Cells transfected with CD1b-M1 were fixed, sectioned, labelled with anti-CD1b antibodies, revealed with a gold-coupled secondary antibody and observed by electron microscopy. **b** Cells transfected with CD1b-M1 were fixed, embedded in Epon resin, stained and sectioned. The nucleus is indicated (N), as well as the location of the nuclear envelope (arrowheads). Scale bar: 1 μm. In the two instances shown, the endoplasmic reticulum collapsed on the nuclear envelope with which it connected in multiple points

In order to assess the effect of Cisd2 on the ER morphology at the ultrastructural level, we expressed in HEK cells the CD1b-M1 chimera, and used a specific antibody to recognize the CD1b extracellular domain by cryo-immuno electron microscopy. In cells where expression of CD1b-M1 was detected, instead of being widely deployed in the cytosol, the ER network often appeared to partially collapse onto the nuclear envelope (arrowheads) with which it connected on multiple points (Fig. 5a). This was also visible in sections of Epon-embedded cells where a better contrast is obtained, although the presence of the CD1b-M1 cannot be assessed (Fig. 5b).

**Fig. 6:**
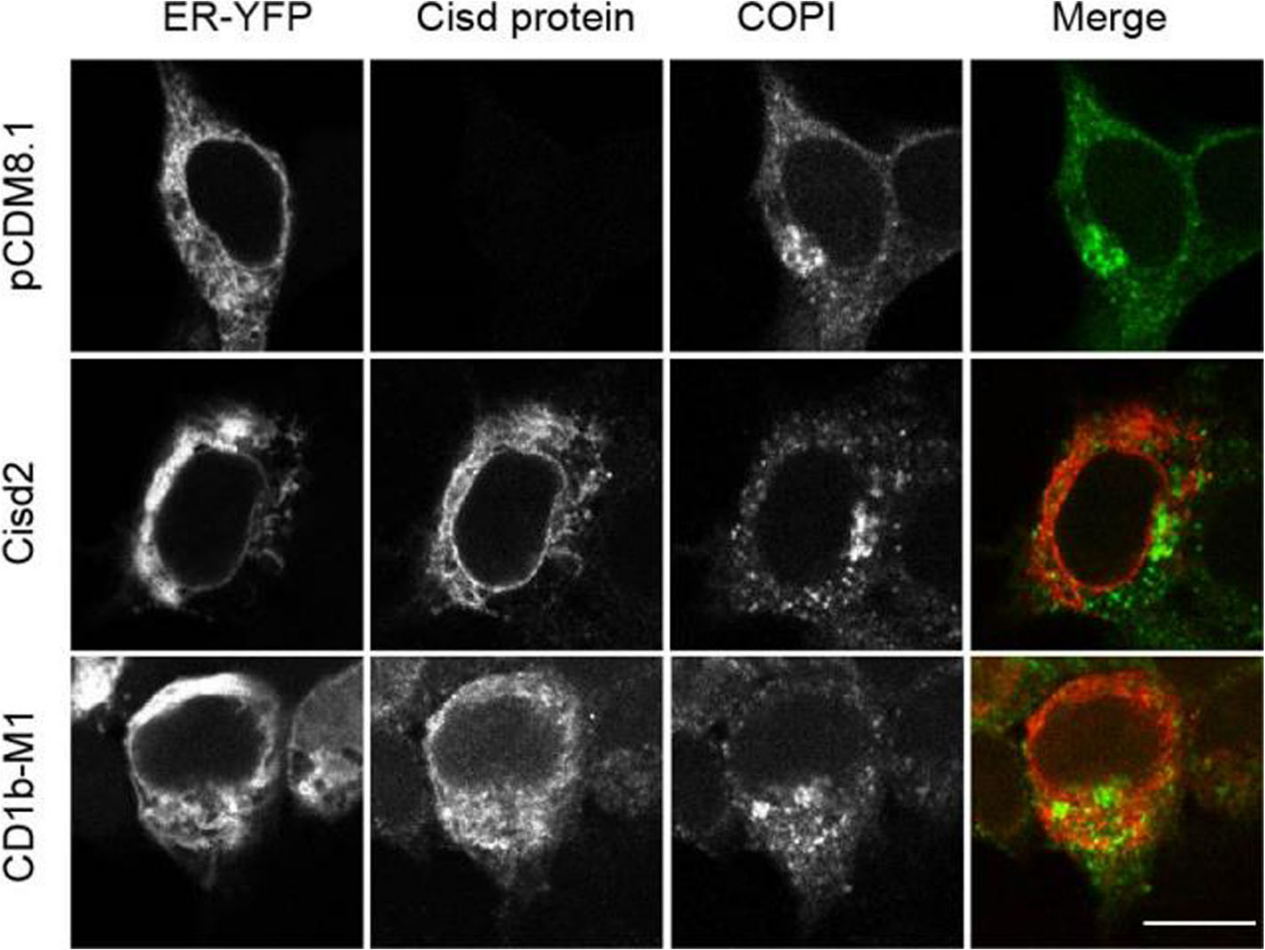
The distribution of COPI is not modified in cells overexpressing Cisd2 or CD1b-Cisd2. Cells were transfected to express ER-YFP together with Cisd2, CD1b-M1 or control plasmid. Each cell was then stained with anti-Cisd2 antibodies (red in merged picture) and anti-COPI antibodies (green). All pictures were taken with a confocal microscope (LSM800, Zeiss). Scale bar: 10 μm. The distribution of COPI was not modified by the overexpression of Cisd2

Since Cisd2 has both the ability to interact with COPI and to modify the structure of the ER, we further studied if high levels of Cisd2 altered the intracellular distribution of COPI. For this, we first selected and characterized recombinant antibodies that recognize the fully assembled COPI complex by immunofluorescence [15, 16]. We then used these antibodies to assess the localization of COPI in cells expressing either Cisd2, or a CD1b-tagged Cisd2. We did not observe in these cells a significant change in the localization of COPI: like in mock-transfected cells, COPI was localized mostly in the peri-Golgi region, as well as in dots throughout the cytosol (Fig. 6). COPI did not noticeably accumulate in regions where the ER structure appeared abnormally expanded.

## Discussion

This study was aimed at clarifying the mechanisms ensuring intracellular targeting of Cisd1 and Cisd2 to different compartments. Our observations indicate that the N-terminus of Cisd1 and Cisd2 target them to mitochondria and to the endoplasmic reticulum, respectively. In addition, the transmembrane and cytosolic domains of Cisd2 both contain motifs ensuring localization of Cisd2 in the endoplasmic reticulum and preventing its transport along the secretory pathway. Finally, expression of Cisd2 alters strongly the morphology of the endoplasmic reticulum, suggesting that Cisd2 controls the morphology of the ER, similar to the structural role observed for Cisd1 in mitochondria.

Although the C-terminal sequences of Cisd1 (KKET) and Cisd2 (KKEV) are very similar, we observed that only the Cisd2 C-terminus was capable of binding the COPI complex. This observation is in general agreement with a systematic analysis of the efficacy with which KKxx sequences can bind COPI and act as ER-targeting motifs [17]: a valine residue at the C-terminal extremity favors efficient ER targeting, while a threonine on the contrary favors escape to the cell surface. *CISD1* and *CISD2* emerged via gene duplication around the origin of vertebrates [18, 19]. It is remarkable that in all vertebrates the C-terminal residue of Cisd2 (V or L) favors binding to COPI and ER targeting, while that of Cisd1 (T, S or A) does not (Additional file 9). This suggests that the dichotomy between the ER-targeting KKxx motif of Cisd2 and the non-ER targeting motif of Cisd1 was present as soon as Cisd1 and Cisd2 emerged. Why Cisd1 exhibits a KKET sequence with no ER-targeting capacity remains unclear. The KKET sequence may play another role, directing for example interaction with a different set of proteins. If this is the case, we must hypothesize that the corresponding protein(s) is less abundant than COPI, or that it does not bind KKET in vitro, since we failed to observe a protein specifically binding to the KKET sequence. Another hypothesis is based on the proposal made earlier that Cisd2 evolved first, and that Cisd1 was formed later by gene duplication [18]. In this scenario the KKET sequence in Cisd1 may be an inactivated remnant of a motif used by Cisd2 to ensure its ER localization but that became useless in the mitochondria-targeted Cisd1. This interpretation is compatible with the observation that no KKxx motif is observed in Cisd proteins from which Cisd1 and Cisd2 have evolved (Additional file 9), nor in Cisd3 proteins that evolved independently from Cisd, Cisd1 and Cisd2 and are located in mitochondria (Additional file 9) [18]. It is striking to find that global phylogenetic analysis [18, 19] and analysis of a very small ER-targeting motif in Cisd2 both lead to similar conclusions: both indicate that the appearance of Cisd1 and Cisd2 represented a major change in the physiology of Cisd proteins, and stress the common origin as well as the functional differences between Cisd1 and Cisd2.

COPI is typically found attached to the membrane of Golgi stacks, but it has also been found in stacked ER structures named coatomer-rich ER (CRER) where two or more ER cisternae are brought together [20] while COPI accumulates in the space separating the two ER cisternae. Although the role of the CRER has not been established, it was proposed to play a role in transport between the ER and the Golgi, either acting as a region retaining COPI-binding KKxx proteins to prevent their incorporation in ER-to-Golgi COPII-coated transport vesicles, or representing the point where Golgi-to-ER COPI-coated vesicles enriched in KKxx proteins may fuse with the ER [20]. The biogenesis of CRER is also not clear, and a protein endowed with multiple ER-targeting motifs including a COPI-interacting motif with the ability to tether ER membranes could in principle be a candidate to generate extensive CRER. We thus wondered whether expression of Cisd2 may alter the distribution of COPI between the ER and the Golgi apparatus. However, we did not observe a significant redistribution of COPI in cells overexpressing Cisd2, nor did we detect an accumulation of COPI in regions where abnormal ER was formed. The simplest interpretation of our observations is that, like Cisd1 in mitochondria, Cisd2 dimers alter the morphology of the ER by tethering two adjacent ER membranes with no intervention of COPI. This hypothesis is reinforced by the observation that when the Cisd2 cytosolic domain is targeted to the mitochondrial membrane (Cisd12 chimera), it causes an aggregation of mitochondria similar to that created by Cisd1 overexpression. More detailed studies will be necessary to determine the precise role of Cisd2 in establishing the morphology of the ER, in particular using cells where endogenous levels of Cisd2 are higher than the very low level observed in HEK cells.

## Conclusions

Although the Cisd1 and Cisd2 sequences share a very similar structure, Cisd2 contains several unique features that direct it to the endoplasmic reticulum and prevent it from being transported along the secretory pathway. The N-terminus of Cisd2 directs it to the ER. In addition, its TMD and cytosolic COPI-binding KKxx motif ensure its retention in the ER. Like Cisd1, Cisd2 is able to modify the structure of the compartment in which it is targeted. A better knowledge of intracellular targeting motifs present in Cisd1 and Cisd2 should facilitate further analysis of the cellular function of these two proteins .

## Methods

### Cell culture and media

HeLa (a kind gift from Pr. N. Demaurex, University of Geneva) and HEK293T (a kind gift from Pr. M. Foti, University of Geneva) cells (referred to as HEK cells) were grown at 37 °C and 8% CO_2_ in Dulbecco’s modified Eagle’s medium (Gibco, #31966–021) containing 10% fetal bovine serum (Gibco, #10270–106) and 100 μg/ml of penicillin-streptomycin (Gibco, #15140–122). Huh-7 cells (a kind gift from Pr. M. Foti, University of Geneva) were grown in the same conditions and with the same supplements, but in low glucose Dulbecco’s modified Eagle’s medium (Gibco, #21885–025), and HCT116 (a kind gift from Dr. F. Lozupone, Istituto Superiore di Sanita, Roma, Italy) cells in Roswell Park Memorial Institute medium (Gibco, #61870–010). To express various fusion proteins, cells were transfected 2 days before the experiment using polyethylenimine (PEI) as previously described [21].

For immunofluorescence detection we used antibodies recognizing the cytosolic domain of Cisd1(RB251; mouse Fc) [11], the cytosolic domain of Cisd2 (RB253; mouse Fc) [12], the extracellular domain of CD1b (AJ521; mouse Fc) [22], the human giantin (AA341; human Fc) [23] and the human COPI complex (RB498; rabbit Fc) [15, 16]. All antibodies were produced by the Geneva Antibody Facility (https://www.unige.ch/medecine/antibodies/) as scFv-Fc minibodies fused with the indicated Fc portion. All antibodies described in this study can be obtained from the Geneva Antibody Facility.

### Immunofluorescence detection of intracellular proteins

The whole procedure was carried out at room temperature. Transfected HEK cells were fixed with PBS + 4% paraformaldehyde for 30 min, and blocked with PBS + 40 mM ammonium chloride (NH4Cl) (Applichem, #A3661) for 5 min. Cells were then permeabilized in PBS + 0.2% saponin (Sigma, #S7900) for 5 min, washed once (5 min) with PBS + 0.2% BSA (PBS-BSA), and incubated for 30 min with the antibody-containing supernatants. After 3 washes (5 min) with PBS-BSA, cells were incubated for 30 min in PBS-BSA with secondary anti-mouse or anti-human or anti-rabbit IgG conjugated to AlexaFluor 546, 647 or 488 (A-11030, A-21236, A-11029, A-21445, A-21245, 1:400, Life Technologies). After 3 washes (5 min) with PBS-BSA, cells were mounted in Möwiol (Fluka, #33480). Pictures were taken using a Zeiss LSM700/800 confocal microscope, with a 63x oil immersion objective.

### Immunofluorescence detection of surface proteins

To reveal the CD1b fusion proteins present at the cell surface, transfected cells were incubated with the anti-CD1b antibody, followed by Alexa-Fluor-488-coupled anti-mouse-IgG antibody (A-11029, 1:400, Life Technologies), both for 30 min at 4 °C. Cells were then fixed for 30 min at room temperature in PBS containing 4% paraformaldehyde and washed with PBS containing 40 mM NH4Cl. Cells were then permeabilized for 5 min in PBS containing 0.2% saponin and labeled with anti-CD1b in PBS-BSA for 30 min. Finally, cells were incubated for 30 min with the Alexa-Fluor-647-coupled anti-mouse-IgG antibody (Life Technologies, A-21236) before being mounted in Möwiol. Surface and total fluorescence intensities were quantified with ImageJ software (http://rsb.info.nih.gov/ij/). For each condition, 70 individual cells were analyzed.

### Immunodetection of CD1b by western blot

To detect CD1b by western blot, 10 × 10^6^ HEK cells were pelleted and lysed in ice-cold PBS containing 0.5% (v/v) Triton X-100, 120 mM NaCl, 25 mM Tris pH 7.4, and protease inhibitors (aprotinin 10 μg/ml, leupeptin 10 μg/ml, iodoacetamide 1.8 mg/ml and PMSF 18 μg/ml). After centrifugation (15 min 10′000 g) the supernatants were collected and resuspended in non-reducing sample buffer (20.6% (w/v) sucrose, 100 mM Tris pH 6.8, 10 mM EDTA, 0.1% (w/v) bromophenol blue, 4% (w/v) SDS), migrated on 4–20% acrylamide gel (SurePAGE Bis-Tris, Genscript #M00655), and transferred to a nitrocellulose membrane using a dry transfer system for 7 min (iBlot2 Dry blotting system, Invitrogen #IB21001). Membrane was then incubated with primary antibody AJ521 against CD1b’s extracellular domain and then with secondary antibody horseradish peroxidase-coupled goat anti-mouse IgG (BioRad, #170–6516, dilution 1:3000). The signal was revealed by enhanced chemiluminescence (ECL) (Millipore, #WBLUC0500) using a PXi-4 gel imaging systems (Syngene).

### Interaction of KKxx motifs with the COPI complex

Synthetic N-terminally biotinylated peptides were immobilized at saturating concentration on Dynabeads® M-280 Streptavidin (Invitrogen #11205D). COS7 cells were lysed in Hepes-Triton buffer (50 mM Hepes (pH 7.3), 90 mM KCl, 0.5% Triton X100 and protease inhibitors) at 3 × 10^6^ cells/ml. 1 mg of beads (approximately 200 pmoles of peptide) were incubated with 1 ml of COS7 cell lysate. After incubation (2 h at 4 °C), beads were washed 3 times in Hepes-Triton buffer and once in 50 mM Hepes. To analyze the proteins bound to the beads, samples were boiled in reducing sample buffer (20.6% (w/v) sucrose, 100 mM Tris pH 6.8, 10 mM EDTA, 0.1% (w/v) bromophenol blue, 4% (w/v) SDS, 6% (v/v) β- mercaptoethanol) and run on a 4–15% acrylamide gel (Mini-PROTEAN® TGXTM Precast Gel, Biorad #456–1086). The gel was first stained using PageBlue Staining Solution (Thermo Scientific #24620). Then the same gel was stained once again with silver [24].

### Electron microscopy

As previously described [8], for conventional electron microscopy, cells were grown and transfected in 10 cm plastic dishes, fixed with 2% glutaraldehyde buffered with 0.1 M sodium phosphate, pH 7.4, postfixed with osmium tetroxide, stained with uranyl acetate, dehydrated in ethanol and embedded in Epon. After sectioning, the samples were observed in a Morgagni electron microscope (FEI).

For immuno-electron microscopy, as previously described [25], transfected cells were fixed for 15 min in the culture medium containing 2% paraformaldehyde and 0.2% glutaraldehyde. The medium was then aspirated and replaced with Phosphate buffer (100 mM NaPO4, pH 7.4) containing the same fixative and incubated further for 1 h. The cells were then detached and pelleted, the fixative was rinsed out three times with Phosphate buffer and the cells processed for cryosectioning. Briefly, the cell pellet was infiltrated with sucrose and frozen in liquid nitrogen. Frozen sections (45-nm thickness) were cut with a Leica FCS cryotome, transferred to grids, and incubated with anti-CD1b antibodies, then with secondary anti-mouse Fc antibodies coupled to 10 nm-gold particles (Goat anti-mouse IgG, EM.GAM10, BBi Solutions).

## Supplementary Information

**Additional file 1.** Immunofluorescence localization of endogenous Cisd1 and Cisd2 in HEK cells. Cells were stained with antibodies specific for Cisd1 or Cisd2. As a negative control, primary antibodies were omitted. As a positive control, cells were transfected to express high levels of Cisd1 or Cisd2. Scale bar: 10 μm.

**Additional file 2.** Immunofluorescence localization of Cisd1 protein in HeLa, HCT116 and Huh-7 cells. **a.** Cells were transfected with mitochondria-targeted RFP. Immunofluorescence staining was performed using specific antibodies against Cisd1. Endogenous Cisd1 appeared exclusively localized in mitochondria. **b.** Cells were co-transfected to produce both the Cisd1 protein and mitochondria targeted RFP. In cells overexpressing Cisd1, mitochondria were seen to form one major aggregate. Scale bar: 10 μm.

**Additional file 3.**Immunofluorescence localization of Cisd2 protein in HeLa, HCT116 and Huh-7 cells. **a.** Cells were transfected with ER-targeted YFP. Immunofluorescence staining was performed using specific antibodies against Cisd2. Endogenous Cisd2 was not detectable in all three cell types analyzed. **b.** Cells were co-transfected to produce both the Cisd2 protein and ER-targeted YFP. Transfected Cisd2 was colocalized with ER-targeted YFP. In several instances, the structure of the ER appeared perturbed. Scale bar: 10 μm.

**Additional file 4.** Amino acid sequence of Cisd1, Cisd2, Cisd12 and Cisd21 proteins. The transmembrane domain, the CDGSH iron sulfur domain (2Fe-2S) cluster and the KKXX domain are shown in boxes.

**Additional file 5.** Immunofluorescence localization of Cisd chimeric proteins. This figure presents a second panel of pictures obtained as described in the legend to Fig. 1.

**Additional file 6.** Amino acid sequence of CD1b-Cisd2 fusion proteins. Cisd2 sequences are shown in black, CD1b sequences in blue. Transmembrane domains are underlined. Original or mutated KKXX motifs are indicated in red.

**Additional file 7.** Colocalization of CD1b-Cisd2 fusion proteins with the ER and Golgi. This figure presents a second panel of pictures obtained as described in the legend to Fig. 2.

**Additional file 8.** Western blot of CD1b fusion proteins reveals glycan maturation. HEK cells were transfected with the indicated CD1b fusion proteins. Cell lysates were separated in non-reducing conditions on an SDS-PAGE gel, and CD1b revealed with a specific antibody. For each fusion protein the size of the proteins bearing immature glycans is indicated with a dot (•) the size of proteins with mature glycans with a star (*). Mature glycans were detected for CD1b-M1, CD1b-M4 and CD1b-M5, but not for ER-targeted CD1b-KKxx or for CD1b-M1, −M2 or -M3.

**Additional file 9.** C-terminal amino acid sequence of Cisd1, Cisd2, Cisd and Cisd3 proteins in various species. The C-terminal region of Cisd1, Cisd2, Cisd and Cisd3 proteins is indicated for a few representative species. Cisd2 presents a highly conserved KKxx ER retrieval motif with the last residue (leucine or valine) favoring ER targeting. Cisd1 presents a non-functional KKxx ER retrieval motif presumably due to an inappropriate last residue (serine, threonine or alanine). Cisd and Cisd3 exhibit no discernible KKxx motif.

## Data Availability

The datasets used and/or analyzed during the current study are available from the corresponding author on reasonable request.

## Abbrevations

ER: Endoplasmic reticulum
TMD: Transmembrane domain
