# Supplementary material for: Intracellular targeting of Cisd2/Miner1 to the endoplasmic reticulum
Source: BMC Mol Cell Biol. 2021 Sep 30;22:48. doi: 10.1186/s12860-021-00387-1 (PMC8482578; doi:10.1186/s12860-021-00387-1)
Supplement: Supplementary file 1 — Additional file 1. Immunofluorescence localization of endogenous Cisd1 and Cisd2 in HEK cells. Cells were stained with antibodies specific for Cisd1 or Cisd2. As a negative control, primary antibodies were omitted. As a positive control, cells were transfected to express high levels of Cisd1 or Cisd2. Scale bar: 10 μm. [file 12860_2021_387_MOESM1_ESM.pdf]

ADDITIONAL FILES

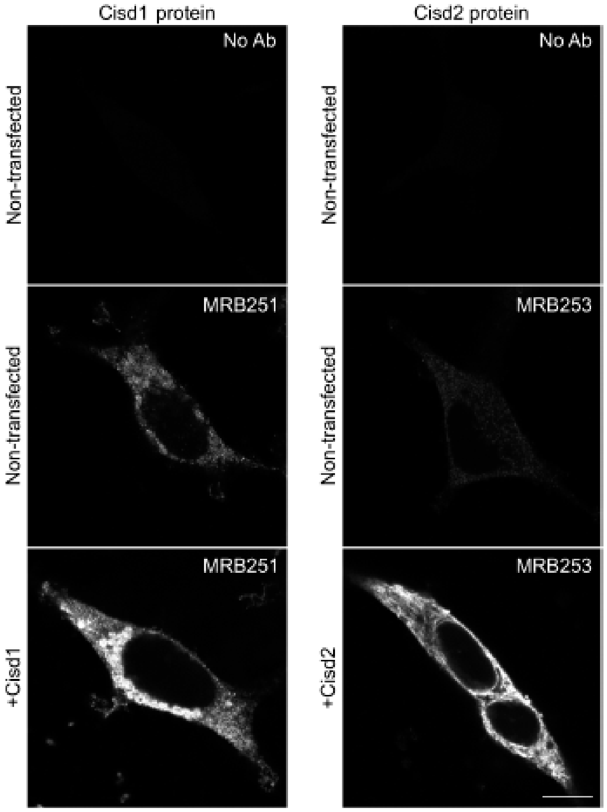

Additional file 1. Immunofluorescence localization of endogenous Cisd1 and Cisd2 in HEK cells. Cells were stained with antibodies specific for Cisd1 or Cisd2. As a negative control, primary antibodies were omitted. As a positive control, cells were transfected to express high levels of Cisd1 or Cisd2. Scale bar: 10 µm.
