# Supplementary material for: Intracellular targeting of Cisd2/Miner1 to the endoplasmic reticulum
Source: BMC Mol Cell Biol. 2021 Sep 30;22:48. doi: 10.1186/s12860-021-00387-1 (PMC8482578; doi:10.1186/s12860-021-00387-1)
Supplement: Supplementary file 2 — Additional file 2. Immunofluorescence localization of Cisd1 protein in HeLa, HCT116 and Huh-7 cells. a. Cells were transfected with mitochondria-targeted RFP. Immunofluorescence staining was performed using specific antibodies against Cisd1. Endogenous Cisd1 appeared exclusively localized in mitochondria. b. Cells were co-transfected to produce both the Cisd1 protein and mitochondria targeted RFP. In cells overexpressing Cisd1, mitochondria were seen to form one major aggregate. Scale bar: 10 μm. [file 12860_2021_387_MOESM2_ESM.pdf]

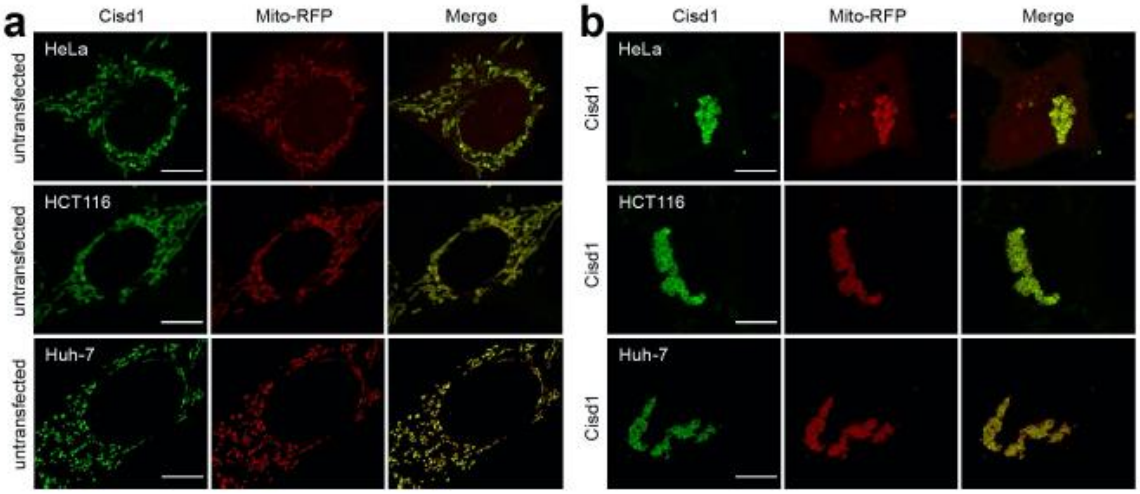

Additional file 2. Immunofluorescence localization of Cisd1 protein in HeLa, HCT116 and Huh-7 cells.

a. Cells were transfected with mitochondria-targeted RFP. Immunofluorescence staining was performed using specific antibodies against Cisd1. Endogenous Cisd1 appeared exclusively localized in mitochondria.

b. Cells were co-transfected to produce both the Cisd1 protein and mitochondria-targeted RFP. In cells overexpressing Cisd1, mitochondria were seen to form one major aggregate. Scale bar: 10 µm.
