# Supplementary material for: Intracellular targeting of Cisd2/Miner1 to the endoplasmic reticulum
Source: BMC Mol Cell Biol. 2021 Sep 30;22:48. doi: 10.1186/s12860-021-00387-1 (PMC8482578; doi:10.1186/s12860-021-00387-1)
Supplement: Supplementary file 3 — Additional file 3.Immunofluorescence localization of Cisd2 protein in HeLa, HCT116 and Huh-7 cells. a. Cells were transfected with ER-targeted YFP. Immunofluorescence staining was performed using specific antibodies against Cisd2. Endogenous Cisd2 was not detectable in all three cell types analyzed. b. Cells were co-transfected to produce both the Cisd2 protein and ER-targeted YFP. Transfected Cisd2 was colocalized with ER-targeted YFP. In several instances, the structure of the ER appeared perturbed. Scale bar: 10 μm. [file 12860_2021_387_MOESM3_ESM.pdf]

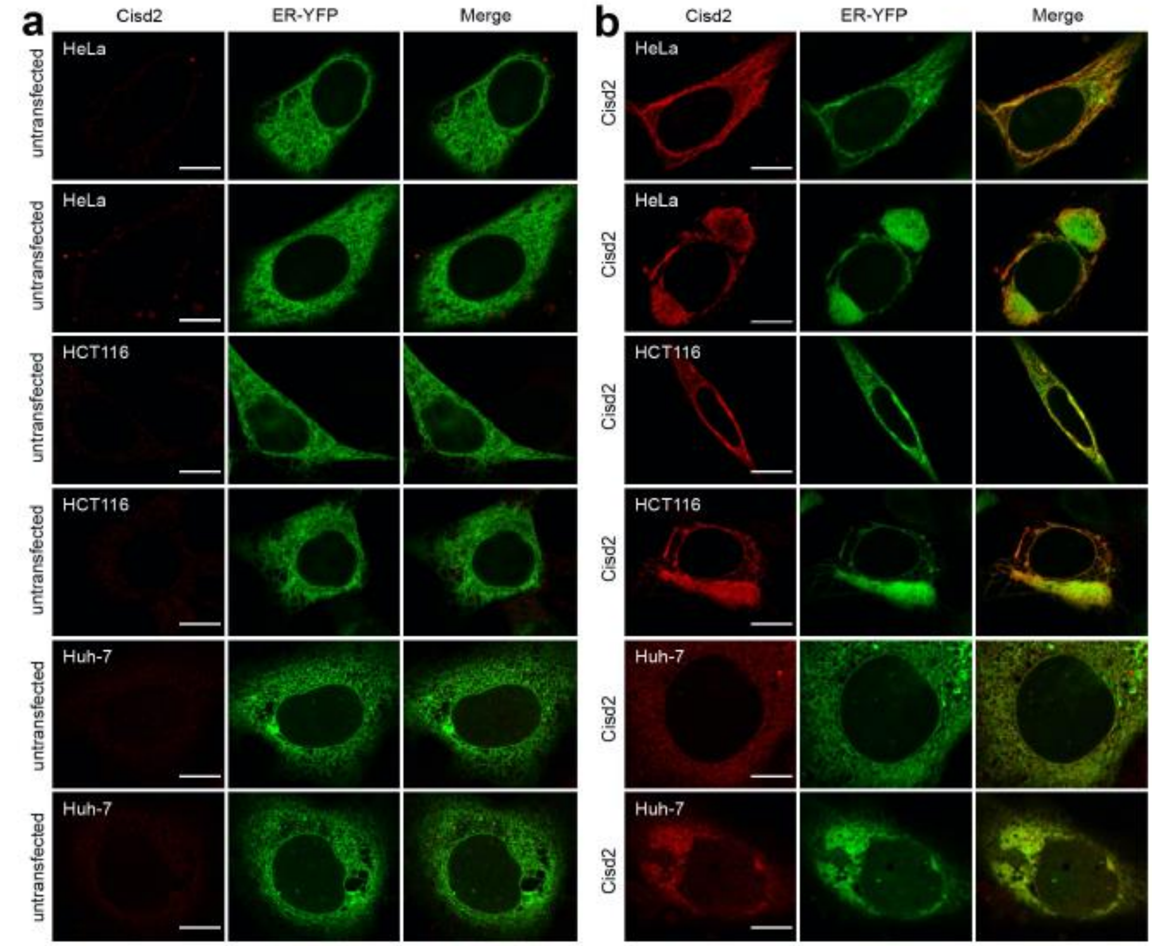

Additional file 3. Immunofluorescence localization of Cisd2 protein in HeLa, HCT116 and Huh-7 cells.

a. Cells were transfected with ER-targeted YFP. Immunofluorescence staining was performed using specific antibodies against Cisd2. Endogenous Cisd2 was not detectable in all three cell types analyzed.

b. Cells were co-transfected to produce both the Cisd2 protein and ER-targeted YFP. Transfected Cisd2 was colocalized with ER-targeted YFP. In several instances, the structure of the ER appeared perturbed. Scale bar: 10 µm.
