# Supplementary material for: Intracellular targeting of Cisd2/Miner1 to the endoplasmic reticulum
Source: BMC Mol Cell Biol. 2021 Sep 30;22:48. doi: 10.1186/s12860-021-00387-1 (PMC8482578; doi:10.1186/s12860-021-00387-1)
Supplement: Supplementary file 4 — Additional file 4. Amino acid sequence of Cisd1, Cisd2, Cisd12 and Cisd21 proteins. The transmembrane domain, the CDGSH iron sulfur domain (2Fe-2S) cluster and the KKXX domain are shown in boxes. [file 12860_2021_387_MOESM4_ESM.pdf]

```
                                                     TM domain
Cisd1     ------------------------MSLTS-SSSVRVEWIAAVTIAAGTAAIGYLAYKRFY    35
Cisd2     MVLESVARIVKVQLPAYLKRLPVPESITGFARLTVSEWLRLLPFLGVLALLGYLAVRPFL    60
Cisd12    ------------------------MSLTS-SSSVRVEWIAAVTIAAGTAAIGYLAYKRFY    35
Cisd21    MVLESVARIVKVQLPAYLKRLPVPESITGFARLTVSEWLRLLPFLGVLALLGYLAVRPFL    60

                                                 CDGSH domain
Cisd1     VKDH-RNKAMINLHIQKDNPKIVHAFDMEDLG-DKAVYCRCWRSKKFPFCDGAHTKHNEE    93
Cisd2     PKKKQQKDSLINLKIQKENPKVVNEINIEDLCLTKAAYCRCWRSKTFPACDGSHNKHNEL   120
Cisd12    VKDH-RNKAMINLKIQKENPKVVNEINIEDLCLTKAAYCRCWRSKTFPACDGSHNKHNEL    94
Cisd21    PKKKQQKDSLINLHIQKDNPKIVHAFDMEDLG-DKAVYCRCWRSKKFPFCDGAHTKHNEE   119

                KKXX sequence
Cisd1     TGDNVGPLIIKKKET                                                108
Cisd2     TGDNVGPLILKKKEV                                                135
Cisd12    TGDNVGPLILKKKEV                                                109
Cisd21    TGDNVGPLIIKKKET                                                134
```

Additional file 4. Amino acid sequence of Cisd1, Cisd2, Cisd12 and Cisd21 proteins. The transmembrane domain, the CDGSH iron sulfur domain (2Fe-2S) cluster and the KKXX domain are shown in boxes.
