# Supplementary material for: Intracellular targeting of Cisd2/Miner1 to the endoplasmic reticulum
Source: BMC Mol Cell Biol. 2021 Sep 30;22:48. doi: 10.1186/s12860-021-00387-1 (PMC8482578; doi:10.1186/s12860-021-00387-1)
Supplement: Supplementary file 5 — Additional file 5. Immunofluorescence localization of Cisd chimeric proteins. This figure presents a second panel of pictures obtained as described in the legend to Fig. 1. [file 12860_2021_387_MOESM5_ESM.pdf]

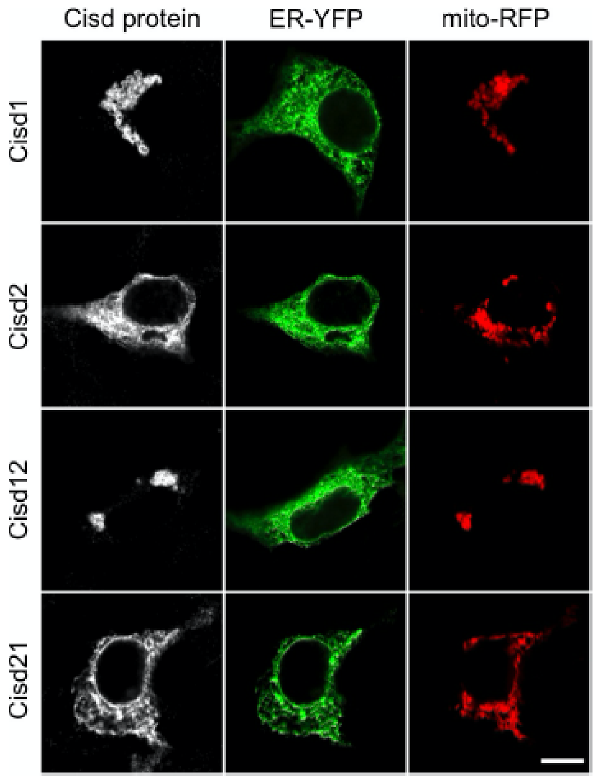

Additional file 5. Immunofluorescence localization of Cisd chimeric proteins. This figure presents a second panel of pictures obtained as described in the legend to figure 1.
