# Supplementary material for: Intracellular targeting of Cisd2/Miner1 to the endoplasmic reticulum
Source: BMC Mol Cell Biol. 2021 Sep 30;22:48. doi: 10.1186/s12860-021-00387-1 (PMC8482578; doi:10.1186/s12860-021-00387-1)
Supplement: Supplementary file 6 — Additional file 6. Amino acid sequence of CD1b-Cisd2 fusion proteins. Cisd2 sequences are shown in black, CD1b sequences in blue. Transmembrane domains are underlined. Original or mutated KKXX motifs are indicated in red. [file 12860_2021_387_MOESM6_ESM.pdf]

```
CD1b-M1     MASRSV-----DILYWRNPTESDSVLESVARIVKVQLPAYLKRLPVPESITGFARLTVSE
            WLRLLPFLGVLALLGYLAVRPFLPKKKQQKDSLINLKIQKENPKVVNEINIEDLCLTKAA
            YCRCWRSKTFPACDGSHNKHNELTGDNVGPLILKKKEV

CD1b-M2     MASRSV-----DILYWRNPTESDSVLESVARIVKVQLPAYLKRLPVPESITGFARLTVSE
            WLRLLPFLGVLALLGYLAVRPFLPRRRSM

CD1b-M3     MASRSV-----DILYWRNPTESDSIVLAIIVPSLLLLLCLALWYMKKKQQKDSLINLKIQ
            KENPKVVNEINIEDLCLTKAAYCRCWRSKTFPACDGSHNKHNELTGDNVGPLILKKKEV

CD1b-M4     MASRSV-----DILYWRNPTESDSIVLAIIVPSLLLLLCLALWYMKKKQQKDSLINLKIQ
            KENPKVVNEINIEDLCLTKAAYCRCWRSKTFPACDGSHNKHNELTGDNVGPLILSSSEV

CD1b-M5     MASRSV-----DILYWRNPTESDSIVLAIIVPSLLLLLCLALWYMKKKQQKDSLINLKIQ
            KENPKVVNEINIEDLCLTKAAYCRCWRSKTFPACDGSHNKHNELTGDNVGPLILKKKEVSSSS
```

Additional file 6. Amino acid sequence of CD1b-Cisd2 fusion proteins.

Cisd2 sequences are shown in black, CD1b sequences in blue. Transmembrane domains are underlined. Original or mutated KKXX motifs are indicated in red.
