# Supplementary material for: Intracellular targeting of Cisd2/Miner1 to the endoplasmic reticulum
Source: BMC Mol Cell Biol. 2021 Sep 30;22:48. doi: 10.1186/s12860-021-00387-1 (PMC8482578; doi:10.1186/s12860-021-00387-1)
Supplement: Supplementary file 7 — Additional file 7. Colocalization of CD1b-Cisd2 fusion proteins with the ER and Golgi. This figure presents a second panel of pictures obtained as described in the legend to Fig. 2. [file 12860_2021_387_MOESM7_ESM.pdf]

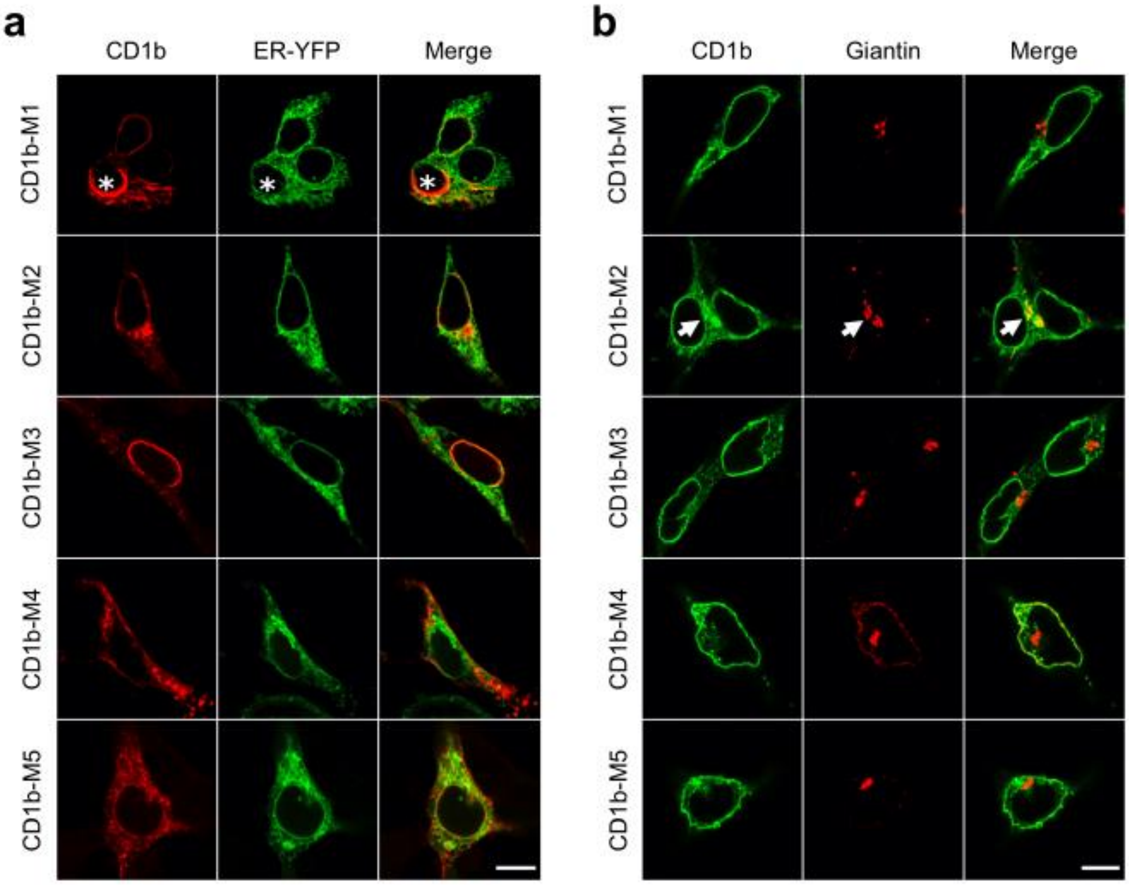

Additional file 7. Colocalization of CD1b-Cisd2 fusion proteins with the ER and Golgi. This figure presents a second panel of pictures obtained as described in the legend to figure 2.
