# Supplementary material for: Intracellular targeting of Cisd2/Miner1 to the endoplasmic reticulum
Source: BMC Mol Cell Biol. 2021 Sep 30;22:48. doi: 10.1186/s12860-021-00387-1 (PMC8482578; doi:10.1186/s12860-021-00387-1)
Supplement: Supplementary file 8 — Additional file 8. Western blot of CD1b fusion proteins reveals glycan maturation. HEK cells were transfected with the indicated CD1b fusion proteins. Cell lysates were separated in non-reducing conditions on an SDS-PAGE gel, and CD1b revealed with a specific antibody. For each fusion protein the size of the proteins bearing immature glycans is indicated with a dot (•) the size of proteins with mature glycans with a star (*). Mature glycans were detected for CD1b-M1, CD1b-M4 and CD1b-M5, but not for ER-targeted CD1b-KKxx or for CD1b-M1, −M2 or -M3. [file 12860_2021_387_MOESM8_ESM.pdf]

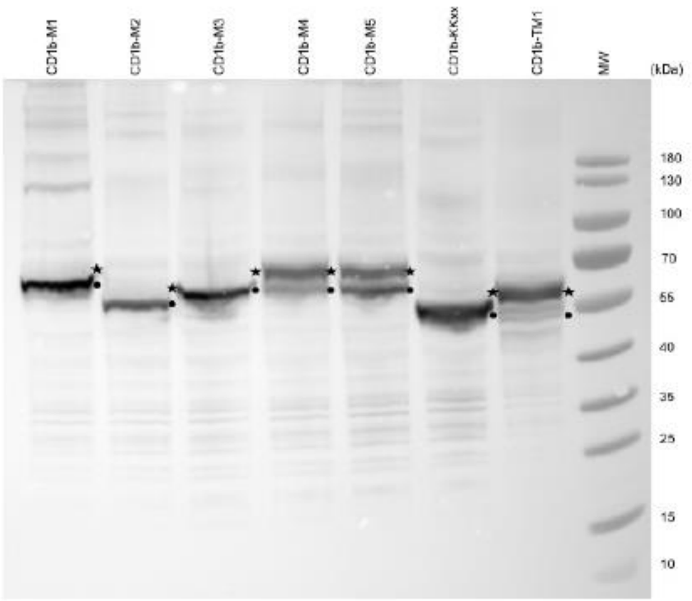


**Additional file 8.** Western blot of CD1b fusion proteins reveals glycan maturation. HEK cells were transfected with the indicated CD1b fusion proteins. Cell lysates were separated in non-reducing conditions on an SDS-PAGE gel, and CD1b revealed with a specific antibody. For each fusion protein the size of the proteins bearing immature glycans is indicated with a dot (•) the size of proteins with mature glycans with a star (*). Mature glycans were detected for CD1b-TM1, CD1b-M4 and CD1b-M5, but not for ER-targeted CD1b-KKxx or for CD1b-M1, -M2 or -M3.
