# Supplementary material for: Intracellular targeting of Cisd2/Miner1 to the endoplasmic reticulum
Source: BMC Mol Cell Biol. 2021 Sep 30;22:48. doi: 10.1186/s12860-021-00387-1 (PMC8482578; doi:10.1186/s12860-021-00387-1)
Supplement: Supplementary file 9 — Additional file 9. C-terminal amino acid sequence of Cisd1, Cisd2, Cisd and Cisd3 proteins in various species. The C-terminal region of Cisd1, Cisd2, Cisd and Cisd3 proteins is indicated for a few representative species. Cisd2 presents a highly conserved KKxx ER retrieval motif with the last residue (leucine or valine) favoring ER targeting. Cisd1 presents a non-functional KKxx ER retrieval motif presumably due to an inappropriate last residue (serine, threonine or alanine). Cisd and Cisd3 exhibit no discernible KKxx motif. [file 12860_2021_387_MOESM9_ESM.pdf]

Cisd1 proteins

Uniprot	Species	Sequence
Q9NZ45	H. sapiens	NEETGDNVGPLIIKKKET
Q91WS0	M. musculus	NEETGDNVGPLIIKKKET
Q3ZBU2	B. taurus	NEETGDNVGPLIIKKKDT
F7AUT1	X. tropicalis	NEETGDNVGPLIIKKKES
Q6PGY7	D. rerio	NQETGDNVGPLIIKRKEA
B5X1R9	S. salar	NQETGDNVGPLIMKRKEA
V9KQ82	C. milii	NDETGDNVGPLIIKKREA

Cisd2 proteins

Uniprot	Species	Sequence
Q8N5K1	H. sapiens	NELTGDNVGPLILKKKEV
Q9CQB5	M. musculus	NELTGDNVGPLILKKKEV
Q05B71	B. taurus	NELTGDNVGPLILKKKEV
Q5I027	X. tropicalis	NELTGDNVGPLILKKKEV
Q7T326	D. rerio	NELTGDNVGPLILKKKTL
B5X8S2	S. salar	NELTGDNVGPLILKKKIL
V9LGN2	C. milii	NELTGDNVGPLIIRKKEV

Cisd proteins

Uniprot	Species	Sequence
Q9VAM6	D. melanogaster	GEHNKQTGDNVGPIVIKK
Q5FC71	C. elegans	GKHNKETGDNVGPLIVKSEKK
T2M4R8	H. vulgaris	NAHNKLSGDNVGPVLLNKK
Q9FLI7	A. thaliana	VKHNKANGDNVGPLLLKKQ

Cisd3 proteins

P0C7P0	H. sapiens	RSERVQKAEVGSPL
B1AR13	M. musculus	KSEQVQKAEVGSPL
G3MWJ2	B. taurus	RSEQVQKAELGSSL
A0A6I8SM75	X. tropicalis	KETHIQNSA
K4FTG2	C. milii	KKNWVQQASLSTCPSRS
Q9U3A1	C. elegans	KTVTDEDKKAGLFD
T2MHK8	H. vulgaris	LEKSVQKCSLGTSIP

Additional file 9. C-terminal amino acid sequence of Cisd1, Cisd2, Cisd and Cisd3 proteins in various species. The C-terminal region of Cisd1, Cisd2, Cisd and Cisd3 proteins is indicated for a few representative species. Cisd2 presents a highly conserved KKxx ER retrieval motif with the last residue (leucine or valine) favoring ER targeting. Cisd1 presents a non-functional KKxx ER retrieval motif presumably due to an inappropriate last residue (serine, threonine or alanine). Cisd and Cisd3 exhibit no discernible KKxx motif.
